# Fat mass and obesity-associated gene polymorphisms, pre-diagnostic plasma adipokine levels and the risk of colorectal cancer: The Japan Public Health Center-based Prospective Study

**DOI:** 10.1371/journal.pone.0229005

**Published:** 2020-02-13

**Authors:** Taiki Yamaji, Motoki Iwasaki, Norie Sawada, Taichi Shimazu, Manami Inoue, Shoichiro Tsugane

**Affiliations:** 1 Epidemiology and Prevention Group, National Cancer Center, Tokyo, Japan; 2 Center for Public Health Sciences, National Cancer Center, Tokyo, Japan; King Saud University, SAUDI ARABIA

## Abstract

Although their functional outcomes remain largely unknown, single nucleotide polymorphisms (SNPs) in the fat mass and obesity-associated gene (*FTO*) may interact with adipokines, especially leptin and adiponectin, to modify the risk of colorectal cancer. We conducted a prospective study of 375 colorectal cancer cases and 750 matched controls to examine the effects of SNPs in the *FTO*, either alone or in interaction with pre-diagnostic plasma adipokine levels. Using a conditional logistic regression model, we obtained odds ratios (ORs) and their 95% confidence intervals (CIs) of colorectal cancer. Seven SNPs in strong linkage disequilibrium demonstrated a similarly positive association with colorectal cancer, and most evidently for rs1558902, rs8050136, rs3751812, and rs9939609 (*P*_trend_ = 0.02). Of interest, we observed a statistically significant interaction of rs8050136 with plasma total adiponectin levels (*P*_interaction_ = 0.03). Compared to non-carriers in the lowest quintile of plasma total adiponectin, A allele carriers in the same quintile showed a considerably elevated risk of colorectal cancer, with a body mass index-adjusted OR of 2.54 (95% CI, 1.36–4.75). This investigation of the interaction between SNPs in the *FTO* and pre-diagnostic plasma adipokine levels has revealed the importance of both genetic and hormonal factors associated with adiposity in colorectal carcinogenesis.

## Introduction

A variety of genome-wide association studies (GWAS) have robustly linked a cluster of single nucleotide polymorphisms (SNPs) in the first intron of the fat mass and obesity-associated gene (commonly abbreviated as *FTO*) to obesity [1–3], which is a well-established risk factor for certain types of malignancies, including colorectal cancer [4]. Subsequently, a number of SNPs within the first intronic region of the *FTO* gene, e.g. rs9939609, have been investigated in relation to the risk of obesity-associated malignancies [5–7], and some studies have reported a significant association with the incidence of breast [8,9], endometrial [10,11], and pancreatic cancers [12]. Although the functional outcome of SNPs in the first intron of the *FTO* gene remains largely unknown, two SNPs, namely rs8050136 and rs1421085, have been reported to modulate expression levels of the *FTO* gene and subsequent clustering of leptin receptor isoform b (LRb), an isoform with the full capacity of leptin signaling [13–15].

Leptin, originally described as the product of the *obese* (*ob*) gene, is predominantly secreted by adipocytes and is accordingly classified as an adipokine [16]. Beyond its well-recognized effects on energy homeostasis [16], leptin has shown the potential to directly stimulate cell proliferation and survival [17]. This effect can be inhibited by adiponectin [18], the most abundant adipokine in the circulation [16]. Given that leptin signaling is mainly mediated via LRb, and that LRb is extensively expressed in epithelial cells of the large intestine [17], SNPs within the first intronic region of the *FTO* gene may alter the impacts of leptin and adiponectin on colorectal carcinogenesis through their plausible connection with LRb.

Here, we conducted a case-control study nested within a large-scale population-based cohort study, the Japan Public Health Center-based Prospective Study (the JPHC Study), to examine the effects of SNPs in the first intron of the *FTO* gene on the incidence of colorectal cancer, either alone or in interaction with circulating levels of leptin and adiponectin.

## Subjects and methods

### Study population

The protocol of the JPHC Study was approved by the institutional review board (IRB) of the National Cancer Center, Tokyo, Japan (Approval number: 2001–021), in accordance with the relevant ethical guidelines for medical research in Japan. The purpose and methods of the study were communicated to the research participants, and consent was obtained implicitly when they responded to the baseline questionnaire and/or donated their blood samples. In the case of blood donation, a list was made in each study area to record those who refused to provide a blood sample. Details of the study have been described elsewhere [19]. In brief, the JPHC study enrolled a total of 68,721 men and 71,699 women who resided in 11 public health center (PHC) areas of 10 prefectures across Japan. Those aged 40–59 years in five PHC areas were enrolled as Cohort I, and those aged 40–69 years in the remaining six PHC areas as Cohort II. Basically, all residents within the targeted age range within each PHC area were involved in the study, with the only exceptions being two PHC areas (Katsushika, Tokyo and Suita, Osaka), for which there were too many residents within the targeted age range for inclusion, and only a portion of these residents were involved.

From 1990 to 1994, the first self-administered questionnaire was distributed to make contact with all enrolled subjects, of whom 53,375 men and 60,086 women replied (response rate: 78% and 84%, respectively). The questionnaire included questions about personal and family medical history as well as lifestyle factors (e.g. smoking, drinking, and dietary habits). Dietary habits were assessed based on a food-frequency questionnaire of 44 items for Cohort I and 52 items for Cohort II.

During almost the same period of time, 18,159 men and 30,852 women donated 10 ml of venous blood for research purposes at the time of their general health examination (acquisition rate: 26% and 43%, respectively). Venous blood was drawn into a vacutainer tube with heparin, and centrifuged to obtain the plasma and buffy coat layer. Samples were cryopreserved at -80 °C until analysis.

For the present investigation of colorectal cancer risk, we excluded one PHC area of Cohort I (Katsushika, Tokyo), for which cancer incidence data were not available, and limited the study subjects to those who had returned the first questionnaire, provided blood samples, and reported no diagnosis of any cancer. Finally, we defined a study population of 14,009 men and 24,377 women, in which this prospective case–control study was nested.

### Case identification and control selection

**Fig 1:**
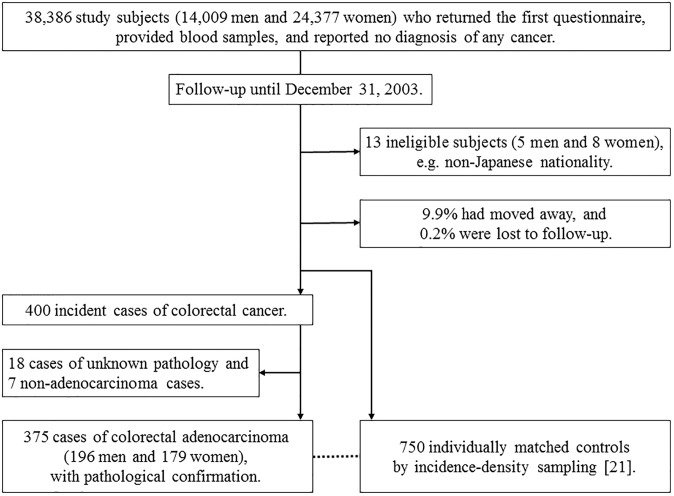
Flow chart of case identification and control selection.

As shown in Fig 1, we followed the study population of 38,386 subjects until December 31, 2003. During that time, 5 men and 8 women were found to be ineligible (e.g. non-Japanese nationality). Changes in residence status, including survival, were identified annually by referring to residential registries of the study area. To confirm the causes of death, we used mortality data from the Ministry of Health, Labor and Welfare. Among the study population, 9.9% had moved away and 0.2% were lost to follow-up during the study period. Incidence data on cancer were collected via two data sources, namely patient records of local major hospitals and population-based cancer registries. Indicators of the completeness of colorectal cancer case-ascertainment conformed to the international standard [20] as follows: information on 5.5% of incident cases first came by way of death certificates (Death Certificate Notification, DCN); 2.2% lacked any detailed information other than a death certificate (Death Certificate Only, DCO); and 94.7% were verified by histological examination (Histological Verification, HV). Up to December 31, 2003, we identified 375 cases of colorectal adenocarcinoma (196 men and 179 women) with pathological confirmation, after excluding 18 cases of unknown pathology and 7 non-adenocarcinoma cases. For each case, two controls were selected using incidence-density sampling [21] from subjects who had no prior history of colorectal cancer at the time the corresponding case was diagnosed with colorectal cancer. Controls were matched for each case with regard to sex, age (within 3 years), date of blood sampling (within 3 months), time since last meal (within 4 hours) and study location (PHC area).

### Case characteristics

All 375 cases of this study were pathologically confirmed as colorectal adenocarcinoma. Of these, 256 subjects had cancer of the colon [International Classification of Diseases for Oncology, Third edition (ICD-O-3) [22] code C180 to C189] and 119 had cancer of the rectum (ICD-O-3 code C199 and C209). Colon cancers were classified into those of the proximal (ICD-O-3 code C180 to C185) or distal colon (ICD-O-3 code C186 and C187). Information on tumor depth was available for 370 of the 375 cases, with 120 tumors of the intramucosal type corresponding to Tis in the TNM classification [23] and 250 of the invasive type corresponding to T1 or more.

### Genotyping of SNPs in the *FTO* gene and measurement of plasma adipokines

To conduct genetic research within the framework of the JPHC Study, we obtained an additional approval from the IRB of the National Cancer Center, Tokyo, Japan (Approval number: 2011–044). Before initiation of the research, we communicated its conduct through our web site, and contacted all living blood donors by mail to provide them with the opportunity to opt-out of participation in the research. Acknowledgement from all eligible subjects was waived by the IRB. We excluded respondents who refused participation in the research, and documented their withdrawal of consent.

For the selection of obesity-related SNPs in the *FTO* gene, we primarily referred to the GWAS catalog (http://www.genome.gov/gwastudies/), and additionally conducted a literature search, resulting in eight SNPs as final candidates, namely rs6499640, rs1421085, rs1558902, rs1121980, rs8050136, rs3751812, rs9939609, and rs9941349. Genomic DNA was extracted from white blood cells in the buffy coat layer using a FlexiGene DNA kit (Qiagen, Hilden, Germany). Buffy coat samples were not available for all 16 pairs (i.e. 48 subjects) in one PHC area of Cohort II (Suita, Osaka). All but 12 buffy coat samples provided a sufficient amount of genomic DNA to perform genotyping. 46 SNPs, including 8 SNPs of the *FTO* gene, were genotyped in a duplicate manner on the BioMark Dynamic Array platform (Fluidigm Corporation, South San Francisco, CA, USA) using the TaqMan SNP Genotyping Assays/ Drug metabolism Genotyping Assays (Applied Biosystems, Foster City, CA) at GeneticLab, Hokkaido, Japan.

Plasma concentrations of leptin were measured using a Human Leptin RIA Kit (LINCO Research, Inc., St. Charles, MO, USA) by the double-antibody radioimmunoassay method at SRL (Tokyo, Japan), which reported intra-assay coefficients of variation of 7.16% to 8.28%. Similarly, plasma concentrations of total and high-molecular-weight (HMW) adiponectin were simultaneously measured using a Human Adiponectin ELISA Kit for Total and Multimers (Sekisui Medical, Co. Ltd, Tokyo, Japan) by the enzyme-linked immunosorbent assay method at Mitsubishi Chemical Medience (Tokyo, Japan), which reported intra-assay coefficients of variation for total and HMW adiponectin of 5.7% and 6.2%, respectively. Plasma samples were available for all study subjects except one.

In genotyping of the *FTO* gene and measurement of plasma adipokines, samples of cases and matched controls were analyzed in the same batch. All laboratory personnel were blinded with respect to case and control status.

### Statistical analysis

First, we compared baseline characteristics between cases and controls using an extension of the Mantel-Haenszel procedure with stratification of matched pairs. We then investigated the association of SNPs in the *FTO* gene and plasma concentrations of adipokines with the incidence of colorectal cancer. Plasma adipokine concentrations were divided into sex-specific quintiles using cutoff points derived from the distribution among controls. Using a conditional logistic regression model, we obtained odds ratios (ORs) and their 95% confidence intervals (CIs) of colorectal cancer with adjustment of matching variables, namely sex, age, date of blood sampling, time since last meal, and study location. In the second model, further adjustment was made for cigarette smoking (never, past, <30, and ≥30 pack-years), alcohol drinking (almost never, 1–3 times/month, <150, 150–299, ≥300 g/week), leisure time physical activity (less than once a week, or once a week or more), family history of colorectal cancer (yes, or no), and plasma concentrations of vitamin D and folate (sex-specific quintiles based on the distribution among controls). Linear trends in the ORs of colorectal cancer were also assessed by assigning ordinal values to each *FTO* genotype or quintiles of respective adipokines. Finally, we evaluated whether SNPs in the first intron of the *FTO* gene interacted with circulating adipokines to modify the risk of colorectal cancer. In this interaction analysis, each *FTO* genotype was dichotomized on the basis of the dominant model, with the first homozygous for the major allele and the second heterozygous and homozygous for the minor allele combined. We then obtained ORs and their 95% CIs of colorectal cancer for 10 combinations of dichotomized *FTO* genotypes and quintiles of plasma adipokines, with reference to the combination of homozygous for the major allele and the lowest quintile of plasma adipokines. Statistical evaluation for multiplicative interaction was made based on the likelihood ratio test with one degree of freedom. An interaction term was created between ordinal variables representing dichotomized *FTO* genotypes and quintiles of plasma adipokines.

Two-sided *P*-values less than 0.05 were regarded as statistically significant. All statistical analyses were conducted using Statistical Analysis System (SAS), Version 9.3 (SAS Institute, Cary, NC, USA).

## Results

### Selected baseline characteristics of cases and controls

Table 1 summarizes selected baseline characteristics of cases and controls. Cases were more likely to be overweight, and also tended to have more experience with smoking and a family history of colorectal cancer than controls. Table 1 also shows plasma concentrations of three adipokines among cases and controls. Although there was no material difference between the two groups, controls tended to have higher plasma concentrations of total and HMW adiponectin than cases.

**Table 1:** Selected baseline characteristics of cases and controls.

Baseline characteristic	Cases		Controls		*P* difference ^c^
Subjects (*n*)	362		709		
Categorical variables, *n* (%)					
Men	185	(51.1)	362	(51.0)	0.98
Ever smoker	155	(43.0)	278	(39.2)	0.12
Ever drinker	171	(47.2)	337	(47.5)	0.88
Leisure time PA (≥1/week)	78	(21.5)	123	(17.3)	0.14
Family history of CRC	9	(2.4)	8	(1.1)	0.05
Continuous variables, adjusted mean					
Age, years ^a^	56.7		56.5		0.72
BMI, kg/m^2^ ^b^	23.7		23.4		0.10
Plasma leptin, ng/ml ^b^	6.20		6.13		0.73
Plasma total adiponectin, μg/ml ^b^	5.98		6.25		0.19
Plasma HMW adiponectin, μg/ml ^b^	2.77		2.94		0.20
Plasma 25-hydroxyvitamin D, ng/ml ^b^	25.5		25.6		0.80
Plasma folate, ng/ml ^b^	8.29		8.29		0.78

Abbreviations: PA, physical activity; CRC, colorectal cancer; BMI, body mass index; HMW, high-molecular-weight.

^a^ Sex-adjusted mean derived from analysis of covariance.

^b^ Sex- and age-adjusted mean derived from analysis of covariance.

^c^ Based on an extension of the Mantel-Haenszel procedure with stratification of matched pairs.

### Association of SNPs in the *FTO* gene with the risk of colorectal cancer

**Fig 2:**
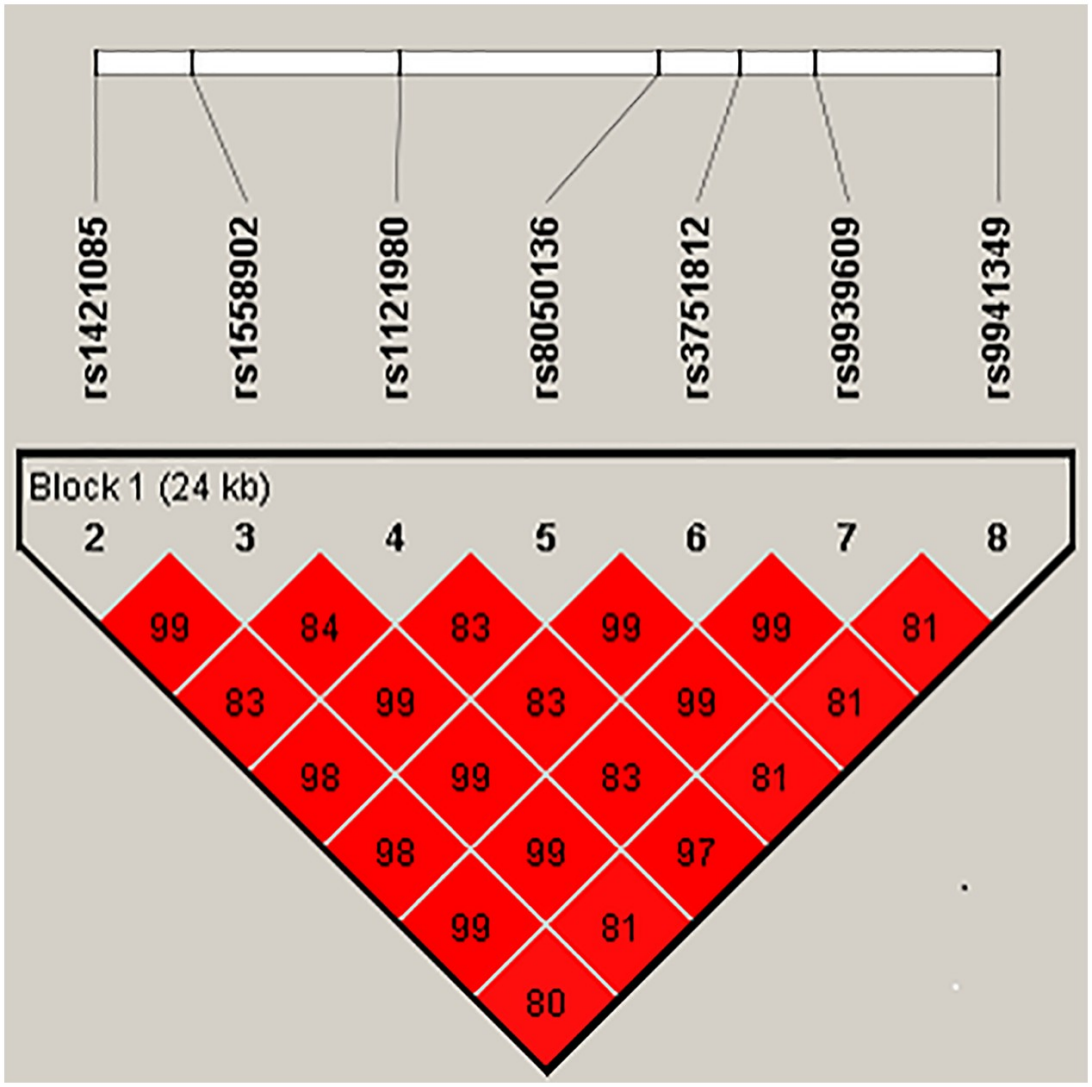
Linkage disequilibrium plot for the analyzed 7 *FTO* SNPs. Linkage disequilibrium (LD) plot generated by Haploview ver. 4.1 (Broad Institute, Cambridge, MA, USA). LD is displayed as pairwise R^2^ values.

Prior to investigating the association between the eight candidate SNPs of the *FTO* gene and the risk of colorectal cancer, we first tested whether genotype frequencies among controls were consistent with those predicted under Hardy-Weinberg equilibrium (HWE) using the χ2 test statistics, and found no inconsistency except for rs6499640 (*P*_HWE_ = 0.01). We then conducted a haplotype analysis excluding rs6499640, and found that the remaining seven SNPs were in strong linkage disequilibrium with R^2^> 0.8 (Fig 2). As highly expected, all seven SNPs in the *FTO* gene demonstrated a similar positive association with the incidence of colorectal cancer (Table 2), and most evidently for rs1558902, rs8050136, rs3751812, and rs9939609 (*P*_trend_ = 0.02). In the case of rs8050136, carriers of the obesity risk A allele had a 36% elevated risk for colorectal cancer (95% CI, 2–83%) compared to non-carriers.

**Table 2:** Association of SNPs in the *FTO* gene with the risk of colorectal cancer.

SNP/ Genotype	Frequency (%)	Controls (*n*)	Cases (*n*)	Model 1 ^a^		Model 2 ^b^	
				OR	(95%CI)	OR	(95%CI)
rs1421085							
TT	66.9	446	212	1.00	(reference)	1.00	(reference)
TC	29.1	194	111	1.19	(0.89–1.60)	1.29	(0.95–1.75)
CC	4.1	27	20	1.53	(0.84–2.81)	1.69	(0.90–3.20)
*P*_trend_^c^					0.09		0.03
TC+CC				1.23	(0.93–1.63)	1.33	(0.99–1.79)
rs1558902							
TT	67.3	449	212	1.00	(reference)	1.00	(reference)
TA	28.6	191	112	1.22	(0.91–1.64)	1.31	(0.97–1.78)
AA	4.1	27	20	1.55	(0.84–2.84)	1.71	(0.90–3.22)
*P*_trend_^c^					0.07		0.02
TA+AA				1.26	(0.95–1.67)	1.36	(1.01–1.82)
rs1121980							
GG	62.5	417	197	1.00	(reference)	1.00	(reference)
GA	31.9	213	124	1.22	(0.91–1.63)	1.28	(0.95–1.73)
AA	5.6	37	23	1.32	(0.75–2.30)	1.43	(0.80–2.55)
*P*_trend_^c^					0.13		0.06
GA+AA				1.23	(0.94–1.63)	1.30	(0.98–1.73)
rs8050136							
CC	67.3	449	212	1.00	(reference)	1.00	(reference)
CA	28.6	191	112	1.22	(0.91–1.64)	1.32	(0.97–1.79)
AA	4.1	27	20	1.55	(0.85–2.85)	1.72	(0.91–3.24)
*P*_trend_^c^					0.06		0.02
CA+AA				1.26	(0.95–1.67)	1.36	(1.02–1.83)
rs3751812							
GG	67.2	448	212	1.00	(reference)	1.00	(reference)
GT	28.8	192	112	1.21	(0.91–1.63)	1.31	(0.97–1.78)
TT	4.1	27	20	1.55	(0.84–2.85)	1.71	(0.91–3.24)
*P*_trend_^c^					0.07		0.02
GT+TT				1.25	(0.95–1.66)	1.36	(1.01–1.82)
rs9939609							
TT	67.3	448	212	1.00	(reference)	1.00	(reference)
TA	28.7	191	111	1.21	(0.90–1.62)	1.31	(0.96–1.78)
AA	4.1	27	20	1.54	(0.84–2.83)	1.70	(0.90–3.22)
*P*_trend_^c^					0.07		0.02
TA+AA				1.25	(0.94–1.66)	1.35	(1.01–1.82)
rs9941349							
CC	63.4	422	197	1.00	(reference)	1.00	(reference)
CT	31.2	208	124	1.27	(0.95–1.69)	1.33	(0.98–1.79)
TT	5.4	36	22	1.33	(0.75–2.36)	1.45	(0.80–2.63)
*P*_trend_^c^					0.09		0.04
CT+TT				1.28	(0.97–1.69)	1.34	(1.01–1.79)

Abbreviations: SNP, single nucleotide polymorphism; OR, odds ratio; CI, confidence interval.

^a^ Adjusted for matching variables, namely sex, age, date of blood sampling, time since last meal, and study location.

^b^ Model 1 + cigarette smoking, alcohol drinking, leisure time physical activity, family history of colorectal cancer, and plasma concentrations of vitamin D and folate.

^c^ Linear trends were assessed by assigning ordinal values to each *FTO* genotype.

### Association of pre-diagnostic plasma adipokine levels with the risk of colorectal cancer

Table 3 shows the association between plasma concentrations of the three adipokines and the risk colorectal cancer. Plasma leptin levels were positively associated with the incidence of colorectal cancer (*P*_trend_ = 0.04), with a multivariate adjusted OR of colorectal cancer for the highest compared to the lowest quintile of 1.80 (95% CI, 1.12–2.88). In contrast, a non-linear inverse association was suggested for total and HMW adiponectin (*P*_trend_ = 0.10 and 0.13, respectively): using the first quintile as reference, the second presented a decrease in the multivariate adjusted OR of colorectal cancer, whereas the third to fifth presented no further decline. Additional adjustment for BMI considerably attenuated the associations of plasma adipokines with the risk of colorectal cancer, but an elevated risk was still statistically significant for plasma leptin levels. Compared to the lowest quintile of plasma leptin, the highest demonstrated a statistically significant increase in the BMI-adjusted OR of colorectal cancer (OR = 1.77; 95% CI, 1.00–3.16; *P* = 0.04).

**Table 3:** Association of pre-diagnostic plasma adipokine levels with the risk of colorectal cancer.

Adipokine	Controls (*n*)	Cases (*n*)	Model 1 ^a^		Model 2 ^b^		Model 3 ^c^	
			OR	(95%CI)	OR	(95%CI)	OR	(95%CI)
Leptin								
Quintile 1	118	49	1.00	(reference)	1.00	(reference)	1.00	(reference)
Quintile 2	162	81	1.25	(0.80–1.97)	1.34	(0.84–2.12)	1.39	(0.85–2.25)
Quintile 3	137	62	1.12	(0.70–1.79)	1.19	(0.73–1.93)	1.21	(0.72–2.06)
Quintile 4	143	71	1.22	(0.77–1.93)	1.20	(0.74–1.93)	1.21	(0.70–2.08)
Quintile 5	149	98	1.63	(1.03–2.58)	1.80	(1.12–2.88)	1.77	(1.00–3.16)
*P*_trend_ ^d^				0.05		0.04		0.15
Total adiponectin								
Quintile 1	141	88	1.00	(reference)	1.00	(reference)	1.00	(reference)
Quintile 2	141	62	0.71	(0.47–1.07)	0.67	(0.44–1.03)	0.68	(0.44–1.05)
Quintile 3	142	87	0.97	(0.65–1.46)	1.02	(0.67–1.54)	1.05	(0.68–1.60)
Quintile 4	141	64	0.67	(0.44–1.04)	0.72	(0.46–1.13)	0.75	(0.47–1.19)
Quintile 5	144	60	0.64	(0.42–0.97)	0.65	(0.42–1.00)	0.68	(0.43–1.08)
*P*_trend_ ^d^				0.05		0.10		0.21
HMW adiponectin								
Quintile 1	139	84	1.00	(reference)	1.00	(reference)	1.00	(reference)
Quintile 2	142	67	0.78	(0.51–1.18)	0.78	(0.51–1.20)	0.78	(0.51–1.20)
Quintile 3	143	78	0.86	(0.57–1.30)	0.91	(0.60–1.38)	0.93	(0.61–1.42)
Quintile 4	142	74	0.81	(0.53–1.22)	0.85	(0.55–1.31)	0.87	(0.56–1.35)
Quintile 5	143	58	0.64	(0.42–0.98)	0.67	(0.43–1.02)	0.71	(0.45–1.11)
*P*_trend_ ^d^				0.08		0.13		0.25

Abbreviations: OR, odds ratio; CI, confidence interval; HMW, high-molecular-weight; BMI, body mass index.

^a^ Adjusted for matching variables, namely sex, age, date of blood sampling, time since last meal, and study location.

^b^ Model 1 + cigarette smoking, alcohol drinking, leisure time physical activity, family history of colorectal cancer, and plasma concentrations of vitamin D and folate.

^c^ Model 2 + BMI.

^d^ Linear trends were assessed by assigning ordinal values to quintiles of respective adipokines.

### Interaction between SNPs in the *FTO* gene and pre-diagnostic plasma adipokine levels on colorectal cancer risk

Finally, we investigated the interaction between SNPs in the *FTO* gene and plasma adipokine levels on colorectal cancer risk. In Table 4, we presented the results of rs8050136 only, because rs8050136 and the other six SNPs were in strong linkage disequilibrium, and all demonstrated closely similar results. Compared to non-carriers in the lowest quintile of leptin, carriers of the obesity risk A allele in the highest quintile had a substantially increased risk of colorectal cancer (multivariate adjusted OR = 2.55; 95% CI, 1.21–5.39), although the interaction was not statistically significant (*P*_interaction_ = 0.30). Of interest, we observed a statistically significant interaction of rs8050136 with total adiponectin (*P*_interaction_ = 0.03), but only a marginal interaction with HMW adiponectin (*P*_interaction_ = 0.16). Compared to non-carriers in the lowest quintile of total adiponectin, carriers of the obesity risk A allele in the same quintile showed a considerably elevated risk of colorectal cancer with the multivariate adjusted OR of 2.54 (95% CI, 1.36–4.75), while the corresponding OR was 2.18 (95% CI, 1.14–4.16) for HMW adiponectin.

**Table 4:** Interaction between SNPs in the *FTO* gene and pre-diagnostic plasma adipokine levels on colorectal cancer risk.

Adipokine	rs8050136								*P*_interaction_^b^
	CC				CA+AA				
	Controls (*n*)	Cases (*n*)	OR ^a^	(95%CI)	Controls (*n*)	Cases (*n*)	OR ^a^	(95%CI)	
Leptin									0.30
Quintile 1	75	33	1.00	(reference)	33	16	1.18	(0.51–2.42)	
Quintile 2	107	49	1.21	(0.66–2.20)	50	29	1.60	(0.80–3.19)	
Quintile 3	86	37	1.04	(0.54–1.99)	42	23	1.54	(0.72–3.27)	
Quintile 4	88	41	1.02	(0.52–1.96)	49	24	1.31	(0.62–2.78)	
Quintile 5	93	51	1.35	(0.67–2.72)	44	40	2.55	(1.21–5.39)	
Total adiponectin									0.03
Quintile 1	96	44	1.00	(reference)	41	41	2.54	(1.36–4.75)	
Quintile 2	92	36	0.83	(0.47–1.47)	39	24	1.50	(0.75–3.00)	
Quintile 3	84	57	1.67	(0.97–2.88)	46	25	1.38	(0.71–2.68)	
Quintile 4	85	37	0.98	(0.54–1.77)	50	21	1.09	(0.54–2.19)	
Quintile 5	92	37	0.85	(0.48–1.50)	42	21	1.23	(0.60–2.52)	
HMW adiponectin									0.16
Quintile 1	92	44	1.00	(reference)	39	36	2.18	(1.14–4.16)	
Quintile 2	94	43	0.99	(0.57–1.70)	41	24	1.37	(0.69–2.74)	
Quintile 3	86	46	1.16	(0.67–2.03)	44	26	1.44	(0.75–2.77)	
Quintile 4	84	44	1.12	(0.63–1.99)	53	24	1.02	(0.53–1.98)	
Quintile 5	93	34	0.74	(0.42–1.31)	41	22	1.35	(0.67–2.71)	

Abbreviations: SNP, single nucleotide polymorphisms; OR, odds ratio; CI, confidence interval; HMW, high-molecular-weight; BMI, body mass index.

^a^ Adjusted for matching variables, namely sex, age, date of blood sampling, time since last meal, and study location, and additionally for cigarette smoking, alcohol drinking, leisure time physical activity, family history of colorectal cancer, plasma concentrations of vitamin D and folate, and BMI.

^b^ Statistical evaluation for multiplicative interaction was made based on the likelihood ratio test with one degree of freedom. An interaction term was created between ordinal variables representing dichotomized *FTO* genotypes and quintiles of plasma adipokines.

## Discussion

In this prospective case-control study, we observed a statistically significant association of SNPs in the *FTO* gene and plasma leptin levels with the incidence of colorectal cancer. Further, as a representative SNP within the first intronic region of the *FTO* gene, rs8050136 interacted with circulating levels of total adiponectin to modify the risk of colorectal cancer. These findings underline the profound involvement of both genetic and hormonal factors related to adiposity in colorectal carcinogenesis.

Several studies have associated various SNPs in the *FTO* gene with the risk of obesity-associated malignancies (best summarized in [24]), e.g. breast [8,9], endometrial [10,11], and pancreatic cancers [12]. With regard to rs8050136, eight studies have investigated its effect on colorectal [25], endometrial [11, 26], pancreatic [27, 28], prostate [29], thyroid [30], and melanoma skin cancers [31], among which two studies showed a nominally significant association with cancers of the pancreas [28] and thyroid [30] in the main analysis. Only one study of colorectal cancer has been conducted [25]: in a multiethnic cohort with more than 2,000 colorectal cancer cases and 9,000 controls, Lim and colleagues observed significant heterogeneity in the association of rs8050136 with colorectal cancer risk by weight status [25]. Among obese individuals (BMI≥ 30 kg/ m^2^), an increase in the number of obesity-risk alleles tended to show an inverse association with the risk of colorectal cancer, versus a weak positive association among normal-weight or overweight individuals (BMI < 30 kg/m^2^) [25]. The latter finding among normal-weight or overweight individuals might support our observations, because almost all subjects (97%) in our Japanese population had a BMI of less than 30 kg/ m^2^.

Circulating levels of leptin have been evaluated in at least seven prospective studies of colorectal cancer, of which four have shown a positive association [32], consistent with our present study. Nonetheless, a recent meta-analysis of prospective studies suggested a non-significant positive association (OR = 1.472; 95% CI, 0.995–2.177), although the heterogeneity between studies was significant (*P* = 0.001) [32]. Of interest, Stattin and colleagues identified a distinct threshold between the third and fourth quartile of leptin, with an OR for the top vs. three bottom quartiles of 2.28 (95% CI, 1.09–4.76) [33]. This might be analogous to our observation of a threshold between the fourth and fifth quintiles. With regard to total adiponectin, three of a total of seven prospective studies have reported an inverse association with the risk of colorectal cancer, and the aforementioned meta-analysis replicated a significant inverse association (OR = 0.716; 95% CI, 0.606–0.847) without significant heterogeneity (*P* = 0.105) [32]. Although we observed a non-linear inverse association, the association was considerably attenuated by further adjustment for potential confounders with BMI.

Although the functional outcome of SNPs within the first intronic region of the *FTO* gene remains largely unknown, rs8050136 in a regulatory region of the *FTO* gene has been linked to expression of the *FTO* gene and subsequent modulation in leptin receptor signaling [13–15]. Beyond its well-recognized effects on energy homeostasis [16], leptin has shown the potential to directly stimulate cell proliferation and survival [17]. In the present study, we observed a statistically significant interaction between rs8050136 and plasma total adiponectin levels on colorectal cancer risk, rather than plasma leptin levels. According to several experimental studies, adiponectin has the potential to inhibit the leptin-induced signaling cascade [18], and a lower abundance of adiponectin likely confers susceptibility to a carcinogenic effect of leptin [18]. In fact, lower adiponectin levels were related to a prominently elevated risk of colorectal cancer among carriers of the obesity risk A allele for rs8050136, which appears consistent with these experimental observations.

A major strength of this study is its prospective nature, in which information and bio-specimens were collected prior to the diagnosis of colorectal cancer, thereby minimizing the potential influence of reverse causality. Also, our study was conducted in a Japanese population with a relatively homogenous genetic background, which likely obviated the possibility of population stratification.

Our study had a number of limitations. First, our subjects were derived from a cohort of health examinees, and thus might have had more favorable lifestyle habits than those who did not attend such checkups [34]. Caution is therefore required in extrapolating our findings to other populations. Second, the sample size of this study was relatively modest, which might have caused false positive findings by chance. Additional studies in a larger population are therefore required. Third, we only focused on 7 SNPs within the first intronic region of the *FTO* gene. Since the initiation of this genetic study, a number of other *FTO* SNPs have been linked to obesity by GWAS. Such obesity-related SNPs in the *FTO* gene might provide additional evidence for our findings. Finally, evidence concerning the functional outcome of SNPs in the first intron of the *FTO* gene is sparse, and the mechanism of the observed associations is difficult to explain. Further investigation of these effects is clearly warranted.

In conclusion, our investigation of SNPs within the first intronic region of the *FTO* gene, either alone or in interaction with circulating adipokine levels, has shed light on the importance of both genetic and hormonal factors associated with adiposity in colorectal carcinogenesis. Although genetic factors are not modifiable, control of plasma leptin and adiponectin levels might lead to a reduced risk of colorectal cancer even among carriers of risk alleles of the *FTO* gene. The findings of this study add to our understanding of the complexities of the association between obesity and colorectal cancer.
